# Supplementary material for: High sensitivity cameras can lower spatial resolution in high-resolution optical microscopy
Source: Nat Commun. 2024 Oct 15;15:8886. doi: 10.1038/s41467-024-53198-1 (PMC11480200; doi:10.1038/s41467-024-53198-1)
Supplement: Supplementary file 1 — Supplementary Information [file 41467_2024_53198_MOESM1_ESM.pdf]

Supplementary Information

High sensitivity cameras can lower spatial resolution in high-resolution optical microscopy

Henning Ortkrass[1], Marcel Müller[1], Anders Kokkvoll Engdahl[1], Gerhard Holst[2], and Thomas Huser[1]
[1]Biomolecular Photonics, Faculty of Physics, Bielefeld University, 33615 Bielefeld, Germany
[2]Excelitas PCO GmbH, Donaupark 11, 93309 Kelheim, Germany

Modulation Transfer Function (MTF) measurement

The MTF of the detection path of the microscope dependent on the camera was measured with a 60x objective lens and a total magnification of 83.3x. We imaged single 100nm TetraSpeck beads (ThermoFisher T7279) within a field of view of 256x256px² ten times. The stack was Fourier-transformed, reduced to absolute-values and averaged. The resulting image was azimuthally averaged. This protocol was performed twice for different beads and the resulting MTFs were averaged. The final MTF was deconvolved with the 2D-projected shape of the spherical bead. The results of these measurements are shown in Suppl. Fig. 1.

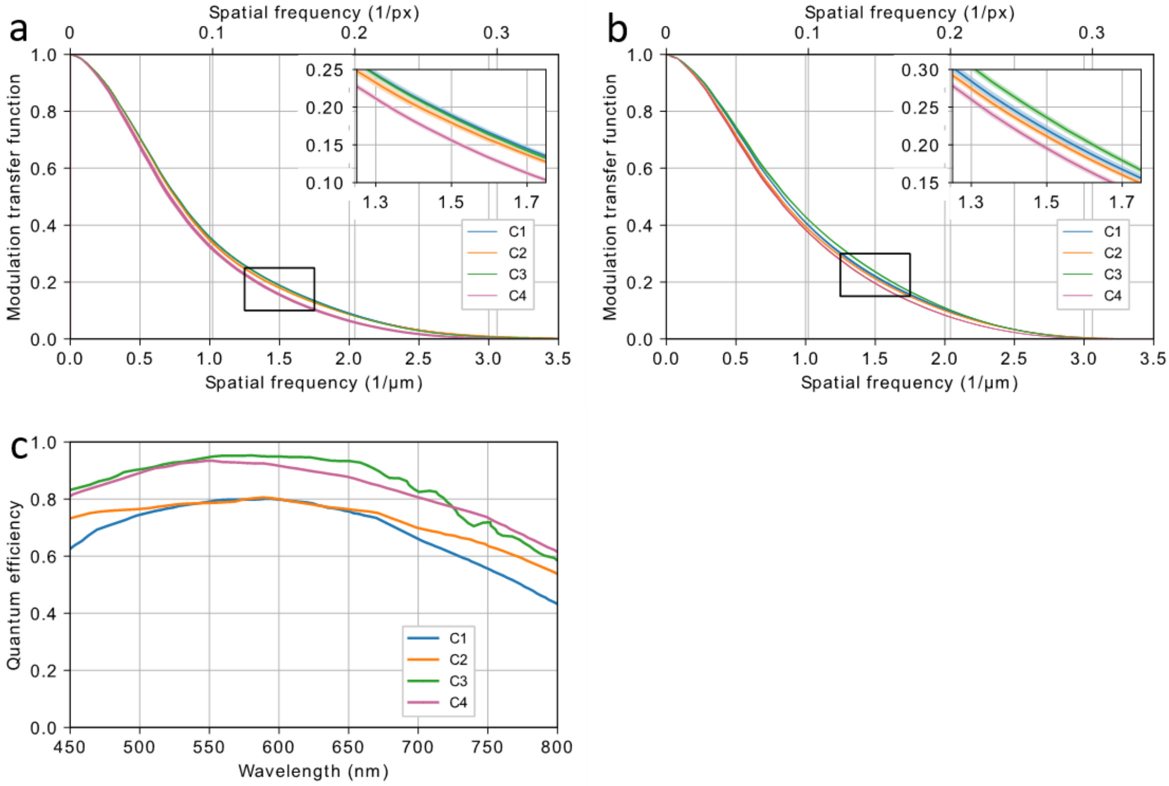

***Supplementary Figure 1**:* Comparison of the modulation transfer function (MTF) of the microscope at a magnification of 83.3x with the front-illuminated (FSI) and back-illuminated (BSI) image sensors**.** The sensor dependent MTF of the system is measured at 555nm (a) and 665nm (b) emission wavelength. The line width corresponds to the standard deviation.The spatial frequency in 1/px corresponds to the camera plane, the spatial frequency in 1/µm to the sample plane. The difference of the MTF of the BSI sensor compared to the FSI sensors is most significant between 1.5/µm and 2/µm. The quantum efficiency (c), that also affects the quality of the super-resolved image reconstruction, is higher for the BSI sensor types.

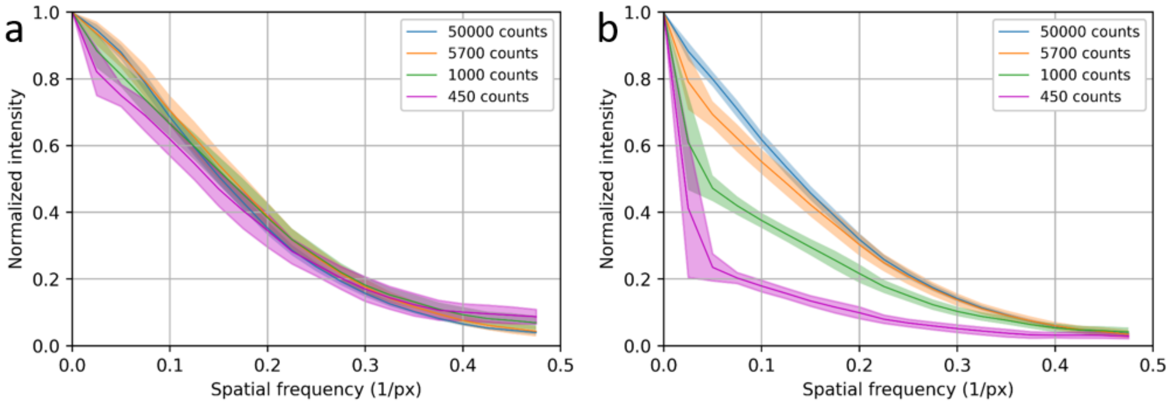

Supplementary Figure 2*:* The MTF of camera C4 at peak intensities between 50 000 counts and 450 counts of the pixel to which the light is focused. For the low contrast image with 450 counts peak intensity, the noise floor is at 116±11 counts. a) Normalized MTF without subtracting the noise floor. b) Normalized MTF, where the noise floor was subtracted before normalization. The line width corresponds to the standard deviation.

Suppl. Fig. 2 shows the normalized MTF of camera C4 calculated from data obtained at different photon counts - measured at the central pixel to which the light was focused. In Fig. 5a the MTFs are shown without subtracting the average noise floor. For the lowest contrast image with 450 photon counts peak intensity, the noise floor is at 116±11 counts, so the signal-to-noise ratio is approx. 4:1. As can be seen from this figure, the noise floor has minimal impact on the MTF taken at high photon counts, which is the condition under which the MTFs of all the other cameras were obtained. Furthermore, by subtracting the noise floor before normalizing the data (see Fig. 5b), the MTFs for all photon count regimes are very similar, which demonstrates that the noise floor has minimal impact on the MTF.

Image resolution measurement

The influence of the camera MTF on the image resolution was tested with additional cameras and with a different test sample. U2OS cells stained with Phalloidin-AF488 were imaged by cameras C1, C5, and C6 (all front-illuminated), as well as C4 (back-illuminated). Since the actin structure of the cells has a high contrast for high spatial frequencies, the image resolution cutoff is less affected by noise. The resulting images (Suppl. Fig. 3) were acquired under identical conditions. The image resolution is evaluated by Fourier ring correlation (FRC). FRC correlates the signal of two images of the same structure, acquired under identical conditions, for different spatial frequencies. If the correlation drops below 0.14, the signal is dominated by noise and the resolution cutoff is reached.[1,2] The resolution cutoff is reduced only by the back-illuminated camera C4 to 245 nm, whereas all the front-illuminated cameras provide a Nyquist limited image resolution of 216 nm.

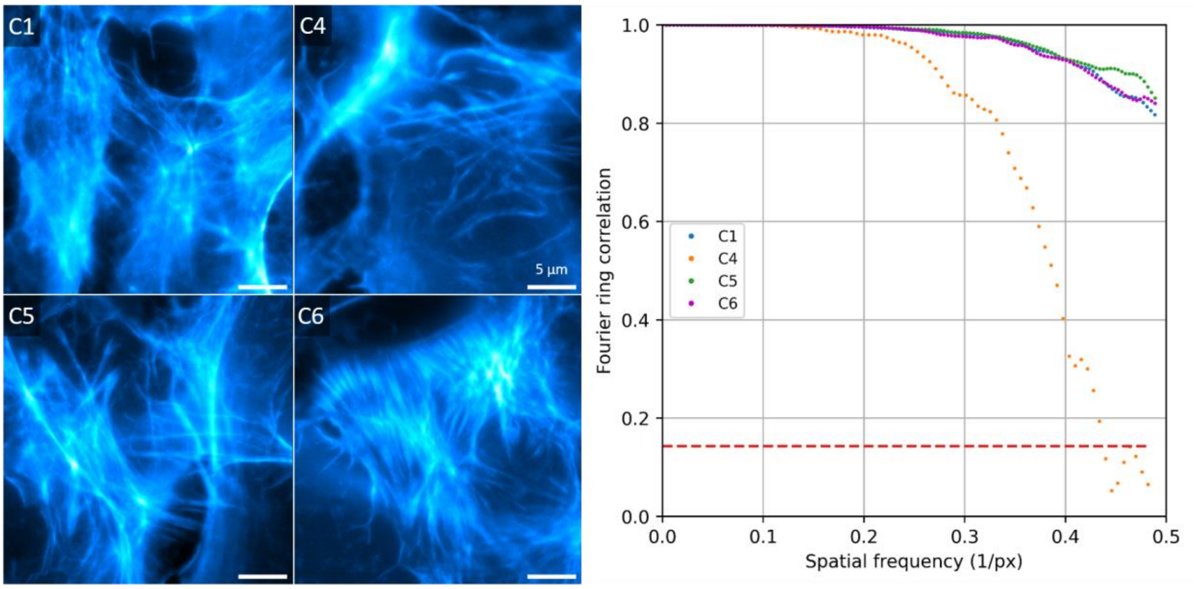

***Supplementary Figure 3**:* Comparison of the image resolution of the microscope at 60x magnification with front-illuminated (FSI) and back-illuminated (BSI) image sensors**.** AF488-Phalloidin stained U2OS cells were imaged with cameras C1, C4, C5 and C6 and the image resolution was measured by Fourier ring correlation (FRC). The images were acquired sequentially by the different cameras with the same exposure time of 100 ms and a laser power adjusted for similar (±5%) average and maximum pixel intensities. The FRC shows that the correlation is far above the 0.14 threshold at the Nyquist frequency for the front-illuminated sensors C1, C5 and C6, corresponding to a Nyquist limited image resolution of 216 nm. The back-illuminated camera C4 provides a resolution of 245 nm under otherwise identical conditions. The spatial resolution of these images is higher than the resolution of the images of membrane- stained cells (Fig. 1 and Suppl. Fig. 5 below) due to the greater extent of high spatial frequencies in the sample structure. The scale bar is 5 µm.

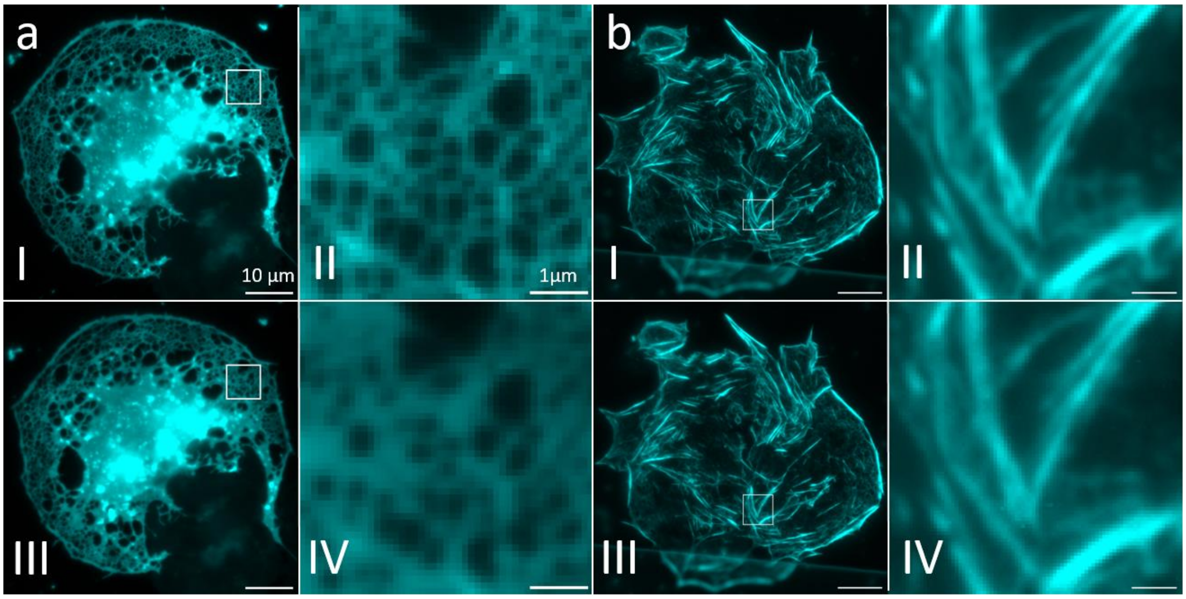

***Supplementary Figure 4**:* The same figure as shown in Fig. 1, but with non-interpolated image insets (aII, aIV, bII, bIV).

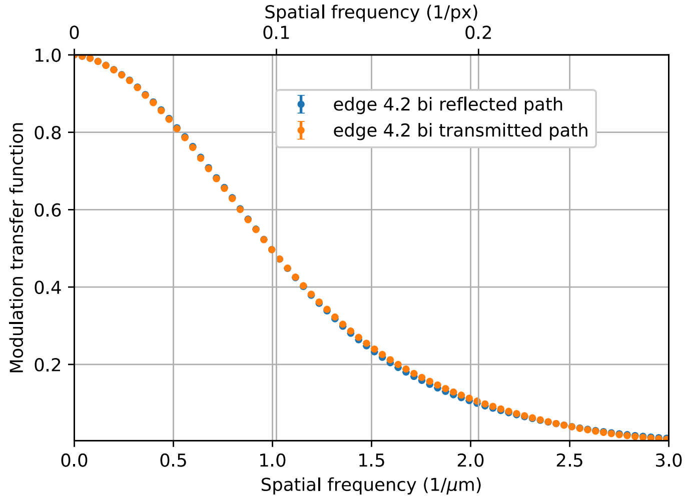

Supplementary Figure 5: The MTF of the microscopes detection path for the transmitted and reflected path of the image splitter cube.

References

1. Banterle, N., Bui, K. H., Lemke, E. A. & Beck, M. Fourier ring correlation as a resolution criterion for super-resolution microscopy. *Journal of Structural Biology* **183**, 363–367 (2013).
2. Nieuwenhuizen, R. P. J. *et al.* Measuring image resolution in optical nanoscopy. *Nat Methods* **10**, 557–562 (2013).
